# The seroconversion history to SARS-CoV-2 in Indigenous people from Brazil – the interplay between exposure, vaccination, and tuberculosis

**DOI:** 10.3389/fimmu.2024.1359066

**Published:** 2024-07-16

**Authors:** Alice Nagai, Renan Barbosa Lemes, José Geraldo Mill, Alexandre Costa Pereira, Rafael Elias Marques, Tábita Hünemeier

**Affiliations:** ^1^ Laboratory of Pathogen Manipulation, Brazilian Biosciences National Laboratory (LNBio), Department of Virology, CNPEM (Brazilian Center for Research in Energy and Materials), Campinas, Brazil; ^2^ Human Population Genomics Laboratory, Biosciences Institute, Department of Genetics and Evolutionary Biology, University of São Paulo, São Paulo, Brazil; ^3^ Health Sciences Center, Department of Physiological Sciences, Federal University of Espírito Santo, Vitória, Brazil; ^4^ Laboratory of Genetics and Molecular Cardiology, Instituto do Coração, Hospital das Clínicas, University of São Paulo, São Paulo, Brazil; ^5^ Department of Genetics, Harvard Medical School, Boston, MA, United States; ^6^ Department of Population Genetics, Institut de Biologia Evolutiva (CSIC/Universitat Pompeu Fabra), Barcelona, Spain

**Keywords:** COVID-19, humoral response, tuberculosis, vaccination, Brazilians, Indigenous

## Abstract

The COVID-19 pandemic caused a significant loss of human lives and a worldwide decline in quality of life. Although our understanding of the pandemic has improved significantly since the beginning, the natural history of COVID-19 and its impacts on under-represented populations, such as Indigenous people from America, remain largely unknown. We performed a retrospective serological survey with two Brazilian Indigenous populations (n=624), Tupiniquim and Guarani-Mbyá. Samples were collected between September 2020 and July 2021: a period comprising the dissemination of SARS-CoV-2 variants and the beginning of COVID-19 vaccination in Brazil. Seroconversions against S and N antigens were assessed using three different commercially available ELISA kits. Samples were also used to assess the prevalence of tuberculosis (TB) in the same population (n=529). Seroconversion against SARS-CoV-2 antigens was considered positive if at least one of the three ELISA kits detected levels of specific antibodies above the threshold specified by the manufacturer. In this sense, we report 56.0% (n=349/623) of seroconverted individuals. Relative seroconversion peaked after introduction of the Coronavac vaccine in February 2021. Vaccination increased the production of anti-S IgG from 3.9% to 48.6%. Our results also indicated that 11.0% (n=46/417) of all individuals were positive for TB. Seroconversion to SARS-CoV-2 was similar between individuals with positive tuberculosis test results to those with negative test results. Most vaccinated individuals seroconverted to SARS-CoV-2, indicating that Coronavac may be as protective in individuals from these indigenous groups as observed in the general Brazilian population. COVID-19 severity was minimal regardless of incomplete vaccine coverage, suggesting that vaccination may not be the only factor protecting individuals from severe COVID-19. Tuberculosis is highly prevalent and not associated with increased seroconversion to SARS-CoV-2.

## 1 Introduction

The pandemic status of COVID-19 was defined in February 2020 by the World Health Organization (WHO) (1). As of April 24th, 2024, COVID-19 has been confirmed in 775,293,630 people and has caused 7,044,637 deaths. Brazil is responsible for roughly 5% of the confirmed cases and 10% of the deaths (2). SARS-CoV-2 is highly transmissible, and COVID-19 has become widespread, reaching even isolated populations (3). COVID-19 reached Indigenous people from South America in 2020, including places such as Amazonas and Pará in Brazil (4–6). The Indigenous population of Brazil includes isolated communities that, due to historical, social, and political reasons, have little access to healthcare (7). Thus, Indigenous in Brazil were included in priority groups for vaccination against SARS-CoV-2 in 2021 (8), together with frontline health and care workers, the elderly, and people with aggravating underlying conditions.

Vaccination, as well as exposure to microorganisms, may induce the production of antibodies that will protect the individual against subsequent infections. These antibodies are usually specific to a certain microorganism; however, non-specific cross-protection may occur, as demonstrated in people vaccinated with the BCG (Bacillus Calmette-Guérin) vaccine who were protected against other infectious diseases (9). In the context of the COVID-19 pandemic, such cross-protection could occur due to trained immunity or due to similarities between protein antigens in SARS-CoV-2 and *Mycobacterium bovis*. The second hypothesis was supported by an immunohistochemistry assay that showed cross hybridization between the envelope (E) protein of SARS-CoV-2 and a consensus protein of *Mycobacterium* sp. Protein sequence analysis using BlastP showed high homology in a 12 amino acids sequence in the region of SARS-CoV-2 E protein used in the immunohistochemistry assay and from a conserved LytRC-terminal domain-containing protein, present in different *Mycobacterium* species (10).

The TB incidence coefficient among the indigenous population residing in the Special Indigenous Health District of Minas Gerais and Espirito Santo achieved 41/100,000 inhabitants (11). This figure is significantly higher than the “End TB Strategy” program’s target of achieving a global TB incidence coefficient lower than 10/100,000 inhabitants by the end of 2035 (12). Considering the high incidence of TB in the indigenous population and the documented homology between proteins of *Mycobacterium* species and SARS-CoV-2, we hypothesized that seroconversion rates to SARS-CoV-2 may exhibit a differential pattern between individuals with and without a history of *M. tuberculosis* infection.

We report a retrospective SARS-CoV-2 serological survey of a large Brazilian admixed Native American population (n=624), composed of Tupiniquim and Guarani-Mbyá ethnicities (13), living in an indigenous reserve in the municipality of Aracruz, Espírito Santo (ES) in the southeast Brazilian coast. We obtained blood samples and clinical data from individuals over a period of 11 months in 2020/2021, before and after the introduction of the Coronavac vaccine in this community. Our results confirm the circulation of SARS-CoV-2 in this population before vaccination, which increased seroconversion for SARS-CoV-2 antigens S and N from 0.96% to 56%. Notably, hospitalization rates due to COVID-19 were remarkably low (n=5/624), indicating that this population may resist severe disease. Moreover, we observed a high prevalence of tuberculosis (11%) which was not associated with higher COVID-19 seroconversion.

## 2 Methods

### 2.1 Overview

We performed an immunological study in 624 individuals from two ethnicities: 555 of Tupiniquim origin and 69 of Guarani-Mbyá. We collected blood samples, sociodemographic and clinical data from all individuals between September 2020 and July 2021. Diabetes mellitus (DM) was defined by fasting glucose >125 mg/dL or DM treatment (insulin or oral hypoglicemiants) and hypertension was defined as presence of clinic blood pressure ≥140/90 mmHg or use of antihypertensive drugs. Almost 80% of the entire population (>18 years) was vaccinated with Coronavac until the end of February/2021.

### 2.2 Seroconversion to SARS-CoV-2

We tested serum samples to detect antibodies against SARS-CoV-2 using three commercially available enzyme-linked immunosorbent assay (ELISA) kits from EuroImmun. These kits targeted anti-S IgA (#EI 2606-9601-A), anti-S IgG (#EI 2606-9601-G), and anti-NCP IgM (#EI 2606-9601-2M). The results were determined by the ratio between the absorbance values of the samples and the calibrator provided in the ELISA kit. Samples with a ratio lower than 0.8 were considered negative, while those with a ratio greater than or equal to 1.1 were considered positive.

### 2.3 Quantification of Interferon-gamma in response to *Mycobacterium tuberculosis* antigens

Whole blood samples were analyzed using the QuantiFERON®-TB Gold Plus (QFT®-Plus) test (Qiagen, #622120) following the manufacturer’s instructions. Briefly, the blood was collected and incubated with control solutions or antigens derived from specific peptides (ESAT-6 and CFP-10) that trigger T lymphocyte responses in individuals infected with *M. tuberculosis*, but not in those vaccinated against tuberculosis with BCG vaccine. The test measured the production of interferon-gamma (IFN-γ), a cytokine released by T cells in response to the *M. tuberculosis* antigens. Positive/negative results were determined based on ratios established by the kit manufacturer, obtained by subtracting the control absorbance values from the test samples. Raw data analysis and result interpretation were performed using QFT Plus Analysis Software (version 2.62).

### 2.4 Statistical analysis

The comparisons between categorical variables were performed with the Chi-Square test with Yates continuity correction under the null hypothesis of independence of the variables using the chisq.test function. The logistic regression was carried out using the Generalized Linear Model available in the glm function, to investigate the relation between COVID-19 and TB status, considering COVID vaccine status as confounding variable. The differences in IFN-γ levels were analyzed with the Mann-Whitney U test. For all the statistical tests, we consider the significant level of p<0.05. Adjusted p-values were estimated considering Benjamini-Hochberg False Discovery Rate (FDR) correction. The statistical analyses were performed using R Studio (version 4.0.4) (14).

## 3 Results

This study evaluated the seroconversion for SARS-CoV-2 antigens and the detection of tuberculosis antigens in two Brazilian Indigenous populations: Tupiniquim and Guarani-Mbyá. The main results are summarized in **Table 1**, including seroconversion status, vaccination (separated by the number of received doses), ethnicity, tuberculosis detection, comorbidities including DM, Systemic arterial hypertension (SAH), gender, age, body mass index (BMI) and hospitalization due to COVID-19.

**Table 1:** Summarized data from the serological survey and the clinical data.

Feature	n° of individuals/total (%, if applicable)
*Seroconversion*	349/623 (56·0)
*COVID-vaccination*	379/623 (60·8)
Doses of COVID-vaccine	
One	34 (9·0)
Two	345 (91·0)
Ethnicity	
Tupiniquim	554/623 (88·9)
Guarani	69/623 (11·1)
*Tuberculosis*	46/417 (11·0)
*Diabetes mellitus (DM)*	71/623 (11·4)
*Systemic arterial hypertension*	205/623 (32·9)
Sex	
Male	251 (40·2)
Female	373 (59·8)
Age (years)	
Lowest	20
Highest	93
…Median	44
BMI (kg/m²)	
Lowest	16·3
Highest	56
…Median	29·8
*Hospitalization*	5/623 (0·8)

**Figure 1:**
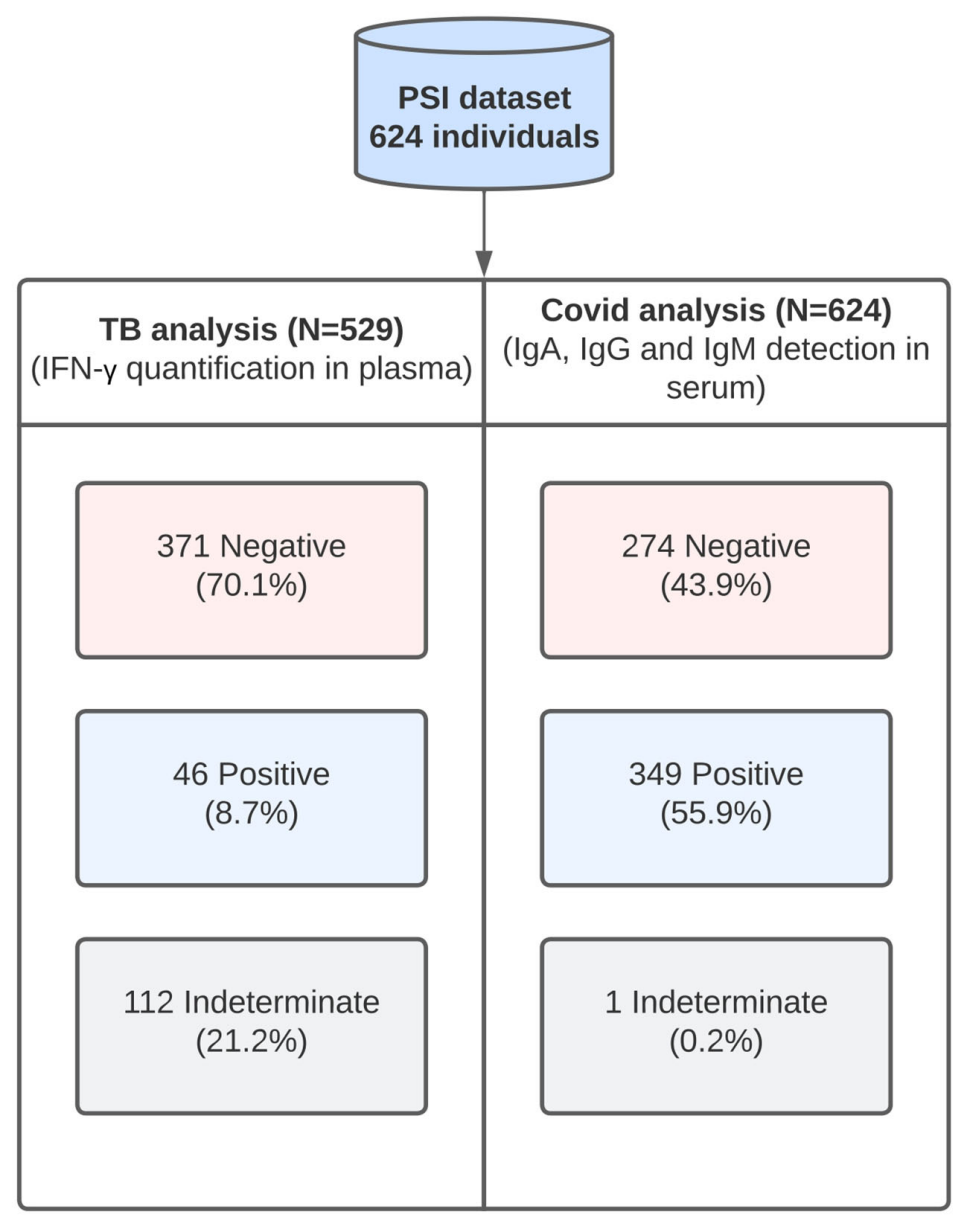
Diagram summarizing the workflow and the main results obtained for tuberculosis (n=529) and COVID-19 (n=624). The status of each individual, i.e., positive, negative or indeterminate, for each analysis was determined based on the thresholds established by the kit. For the tuberculosis analysis, the results were considered positive if either the TB1 or the TB2 tubes were positive. Both TB1 and TB2 tubes contain the CFP-10 and ESAT-6 antigens. In the TB1 tube, these antigens were designed to elicit CD4+ response and in the TB2 tube, an additional set of peptides was included to elicit CD8+ response. For the COVID-19 analysis, the results were positive if any of the immunoglobulins was detected in the sample.

We analyzed serum samples from 624 individuals using ELISA kits to detect either anti-SARS-CoV-2 IgA, IgG or IgM antibodies. In the first approach, a sample was considered positive if at least one of the three targeted antibodies (IgA, IgG, or IgM) exceeded the manufacturer’s designated cut-off value. Alternatively, the results for each antibody were analyzed independently. Of 624 samples tested, 349 (55.9%) were positive in one or more assays and 274 (43.9%) were negative. One indeterminate sample was excluded, totalizing 623 valid samples (**Figure 1**).

Plasma levels of IFN-γ analyzed in 529 samples of blood stimulated with TB infection-specific peptides to indicate T CD4+ (TB1 tube) and T CD8+ (TB2 tube) lymphocyte responses, indicated that 46 individuals were positive (TB+), 371 were negative (TB-), and 112 had an indeterminate status (**Figure 1**). Excluding the indeterminate samples, the percentage of TB+ individuals was 11.0% (**Table 1**), in the period between September 2020 and June 2021.

**Figure 2:**
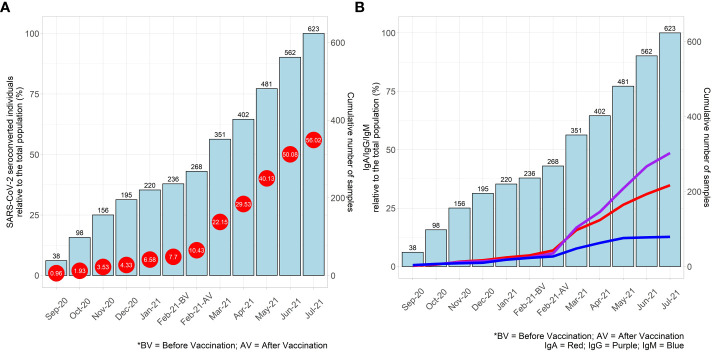
**(A)** Percentage of cumulative seroconversion results to SARS-CoV-2 (represented by red circles) and the corresponding number of cumulative samples collected (represented by blue bars) are shown for the period between September 2020 and July 2021. Seroconversion was considered positive if any immunoglobulin was detected in an individual. The percentages were calculated in relation to the total number of the valid results (n=623). **(B)** Percentage of cumulative positive results for each immunoglobulin against SARS-CoV-2 (IgA - red line, IgG - purple line, IgM - blue line) and the corresponding number of cumulative samples collected (represented by blue bars) are shown for the period between September 2020 and July 2021. The percentages were calculated in relation to the total number of the valid results (n=623).

The history of seroconversion for SARS-CoV-2 antigens is depicted in **Figure 2**. Seroconverted individuals (1.0%) were already observed at the first timepoint, September 2020, gradually increasing up to 7.7% in February 2021, confirming that these Native-American populations were exposed to SARS-CoV-2 before vaccination. With the introduction of the Coronavac (Sinovac) vaccine in late February 2021, we observed a steep rise in the percentage of seroconversion (**Figure 2A**). Before vaccination, the percentages of the antibodies were similar to each other (adjusted p-value = 0.54). After vaccination, IgG and IgA were detected in higher percentages (adjusted p-value < 0.0001) (**Figure 2B**).

Association studies using the Chi-square test with the SARS-CoV-2 seroconversion status of individuals and factors such as vaccination status (adjusted p-value < 0.0001), the number of vaccine doses received, ethnicity, TB status, gender, comorbidities (DM and SAH) indicated that seroconverted individuals were observed at a higher proportion when variables such as two vaccine doses compared to one dose (adjusted p-value = 0.017), Guarani-Mbyá ethnicity compared to Tupiniquim (adjusted p-value = 0.019), or TB+ compared to TB-, were tested. However, we found equal proportions of seroconverted individuals when analyzing variables such as gender (adjusted p-value = 0.97) and comorbidities (DM and SAH) (adjusted p-values = 1; 0.64, respectively).

Regarding vaccination, 379 individuals received at least one dose of the Coronavac vaccine, and 244 individuals were not vaccinated, resulting in 60.8% of vaccinated individuals in the entire study population during the period. Among vaccinated people, 9.0% (n=34) received one dose, and 91.0% (n=345) received two doses (**Table 1**). As expected, a higher percentage of seroconversion was observed in individuals receiving two vaccine doses (80.3%), in comparison to individuals receiving only one dose (58.8%) (p-value = 0.0071).

In the non-vaccinated group, 52 individuals (21.3%) seroconverted, which we attributed to SARS-CoV-2 transmission. The number of Tupiniquim individuals who seroconverted (298 - 53.8%) was statistically similar to those who did not seroconvert (256 - 46.2%) (adjusted p-value = 0.07). However, in the Guarani-Mbyá population, more people seroconverted (51 - 73.9%) (adjusted p-value < 0.0001), likely due to the high percentage of complete vaccination (94.2%), versus 56.7% of vaccinated Tupiniquim, indicating complete vaccination as the main driver of seroconversion in these populations.

As stated before, a higher percentage of SARS-CoV-2 seroconversion was detected in TB+ people (73.9%) when compared to TB- (52.7%) (adjusted p-value = 0.002) (**Supplementary Figure 1**). Since SARS-CoV-2 seroconversion is higher in vaccinated individuals, we repeated the statistical analysis of seroconversion in TB+ and TB- individuals but separately in COVID-19 vaccinated and non-vaccinated individuals. The seroconversion status proportions were similar between non-vaccinated TB+ and TB- individuals (adjusted p-value = 0.36). Therefore, these results showed that vaccination, rather than a TB+ status, influenced seroconversion rates.

One hundred and two individuals were previously tested for COVID-19 during the period, of which 92 were positive. As expected, we observed a higher proportion of seroconversions (88.0%) (adjusted p-value = 0.014) among positive individuals. The logistic regression to evaluate the association of TB+ status and SARS-CoV-2 infection, including individuals that reported a positive antigen test/viral genome detection or seroconverted for SARS-CoV-2 without vaccination, indicated no association (adjusted p-value = 0.57). Furthermore, among the 92 individuals who tested positive for COVID-19, 11 did not seroconvert. Interestingly, four individuals did not seroconvert even after receiving two doses of the vaccine.

The quantification of IFN-γ produced by T lymphocytes in response to tuberculosis antigens (**Supplementary Figure 2**) was compared between individuals that seroconverted or not for SARS-CoV-2. In TB+ individuals, no differences in IFN-γ levels were observed between people who seroconverted or not, regardless of having CD4+ (Mann-Whitney U test adjusted p value = 0.17; **Supplementary Figure 2A**) or CD8+ (Mann-Whitney U test adjusted p value = 0.99; **Supplementary Figure 2B**) lymphocytes as source.

Remarkably, the hospitalization rate due to severe COVID-19 was low (5/623). All hospitalized individuals are Tupiniquim women with ages ranging from 21 to 56. Four individuals were not vaccinated, and one received two vaccine doses. Three individuals required respirators, including the vaccinated patient, who was the youngest and obese (BMI 44 kg/m²).

The separate analysis of each antibody class assessed in the serological tests against SARS-CoV-2 antigens indicated that, in 623 samples, 79 (12.7%) were positive for IgM, 217 (34.8%) were positive for IgA, 303 (48.6%) were positive for IgG. Among positive samples, the detection of two or more classes of antibodies was common and both IgA and IgG were detected in 145 individuals; IgA and IgM were only observed in 3 people; IgG and IgM, in 14 individuals, and finally, all three classes of antibodies were detected in 44 people (**Supplementary Figure 3**). In this group (n=44), 36 were vaccinated with at least one dose and eight individuals were not vaccinated. SARS-CoV-2 infection was reported in six (n=6/8) triple-positive-non-vaccinated individuals using commercially available tests.

**Figure 3:**
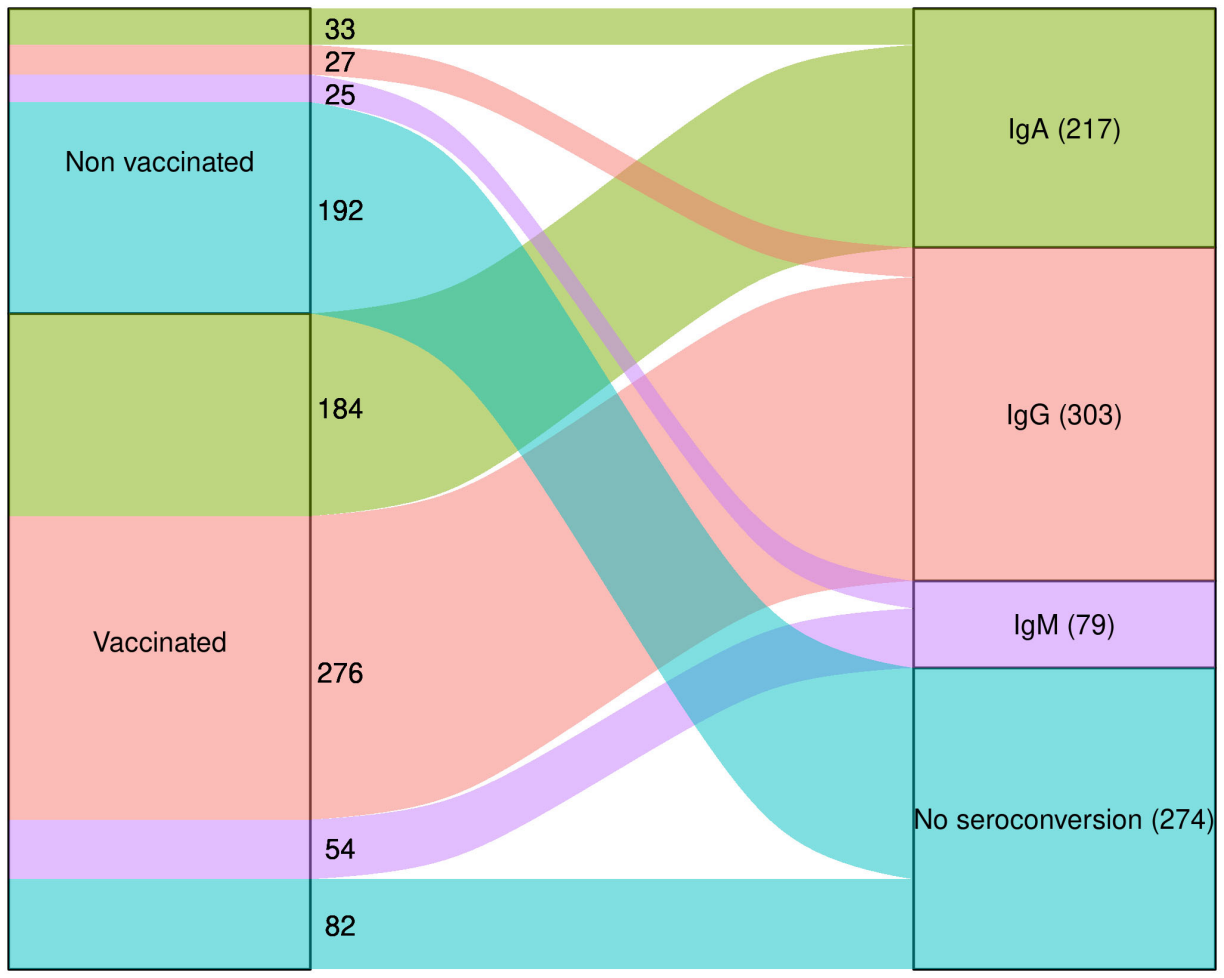
Overview of seroconversion and non seroconversion to SARS-CoV-2. Raw number of individuals that are positive to IgA (green), IgG (salmon), IgM (purple), or negative to all of them are represented separately in the non-vaccinated and vaccinated groups on the left side of the chart, which converge to the total values of the immunoglobulins and non-seroconversion independently of the vaccination status represented on the right side.

In the non-vaccinated group, SARS-CoV-2 seroconverted individuals presented similar proportions (adjusted p-value = 0.54) of positive IgM (25), IgA (33), IgG (27) tests. On the other hand, in the vaccinated group, we detected a lower proportion of positive IgM tests (54 individuals) and increased IgG positive tests (276 individuals) (adjusted p-value < 0.0001) (**Figure 3**). The comparison of seroconversion status in both groups (vaccinated and non-vaccinated) showed that seroconversion is linked to vaccination (adjusted p-value < 0.0001). The proportion of anti-SARS-CoV-2 antibody classes was compared between groups receiving one or two vaccine doses and showed that complete vaccination was associated with a predominant anti-SARS-CoV-2 IgG seroconversion (adjusted p-value < 0.0001). Additionally, anti-SARS-CoV-2 antibody class proportions were similar between ethnicities (p = 0.76).

## 4 Discussion

The impact of COVID-19 in Indigenous populations living in reserves is largely unknown. Among several reasons, the lack of inclusive public health strategies and inherent difficulties to access isolated communities, and to obtain and process samples, have impaired a more significant advance in this field of research. Based on our review of the literature, our study includes the largest number of plasma samples (n=624) from Brazilian Tupiniquim and Guarani-Mbyá individuals living in an indigenous reserve to this date. Our study invariably indicated that the dissemination of SARS-CoV-2, seroconversion, and the association of risk factors with COVID-19 were different in these populations when compared to non-isolated Brazilian populations. In fact, we observed that less than 10% of individuals seroconverted to SARS-CoV-2 from September 2020 to February 2021, before the vaccination program was initiated. This incidence is less than a third the incidence in non-indigenous populations in Pará State, in the north region of Brazil (15), though both regions were considered high risk areas for COVID-19 in 2020-2021 (16).

Importantly, introduction of vaccination in the study population was the main driver of anti-SARS-CoV-2 seroconversion. Generation of neutralizing antibodies against the S protein, mostly IgG, is still considered the main factor in a successful immunization against SARS-CoV-2 (17, 18). It is unknown whether COVID-19 vaccine effectiveness could be similar between the average Brazilian and indigenous populations, and studies addressing this question are still lacking. Unfortunately, our study design, focused on a seroepidemiological assessment of an underrepresented population in Brazil, is not adequate for the assessment of vaccine efficacy, which should focus on the occurrence of infection/disease in vaccinated versus non-vaccinated experimental groups, among other aspects. The emergency of COVID-19 and the obvious risk to human life required that as many people in our study population were vaccinated as fast as possible, which also impaired a vaccine efficacy study impossible. We could not determine if the SARS-CoV-2 Gamma variant of concern (VOC), which emerged in Brazil during the time of sample collection and was neutralizing antibody escape mutant (18), reached the relatively isolated Tupiniquim and Guarani-Mbyá populations included in this study. Although we were unable to assess neutralizing activity in our samples, vaccination induced a significant increase in the proportion of individuals expressing anti-S IgG, which suggests that vaccination induced antibody isotype changes and protective humoral responses against SARS-CoV-2.

Seroconversion for both IgA and IgG was detected in 41.5% of all seroconverting individuals in our study, representing the highest proportion compared to other antibodies detected either for only a specific class or for two or more classes detected in conjunction. Similar results were reported for other Indigenous community from Amazonas, with the highest percentage of both IgA and IgG (4).

We observed an association between seroconversion against SARS-CoV-2 and parameters such as vaccination, number of vaccine doses, and ethnicity. There was no association between seroconversion and gender, which has been reported elsewhere (4, 15, 19). Several risk factors are associated with a more severe course of COVID-19. These factors include chronic comorbidities such as diabetes mellitus (DM) and systemic arterial hypertension (SAH), as well as advanced age, particularly in men, as reported in the literature (4). Therefore, we performed association studies using Qui-Square tests with each of these factors separately, and seroconversion. Surprisingly, our results did not support an association between seroconversion for SARS-CoV-2 and risk factors such as DM and SAH in this population. Individuals with such risk factors may present longer viremia and thus develop stronger adaptive responses to SARS-CoV-2. Although counter intuitive, we hypothesized that seroconversion and risk of severe COVID-19 may be independent variables, especially considering the population evaluated is indigenous and the average BMI is above the Brazilian average (6, 20). Male indigenous individuals aged over 80 years (16) were found to be more susceptible to COVID-19, which is in accordance with the now established association between COVID-19 and advanced age (6). In our study, we were not able to assess the association of advanced age with seroconversion in male individuals, because individuals who were over 80 years old were all female and the mean age of the studied population was 44.

We found no evidence indicating that TB may affect rates of seroconversion for SARS-CoV-2. Although the impact of COVID-19 on this population in terms of morbidity seems discrete, the high prevalence of TB infections confirms that further action to treat and control TB are necessary and urgent. In terms of seroconversion for SARS-CoV-2, we observed similar proportions between TB+ and TB- individuals when we analyzed vaccinated and non-vaccinated individuals separately. Moreover, there was no correlation between seroconversion and TB status, assessed using Logistic Regression, and levels of IFN-γ were comparable between people who seroconverted or not against SARS-CoV-2 antigens. In a study from Torres and colleagues, there was no correlation between tuberculosis and seropositivity against SARS-CoV-2 in non-indigenous population from Pará. Instead, authors report other factors, such as contact with infected people, low income, the level of education and advanced age as risk factors (6).

The hospitalization rate was low (less than 1%) in individuals analyzed in this study. In accordance with our results, a seroepidemiological study performed with Amazonian Indigenous communities reported the majority of COVID-19 cases as being asymptomatic or mild. Authors also reported that mortality associated with disease outbreaks originating in urban areas take longer to reach indigenous communities (5). Furthermore, the “Virgin-soil” hypothesis, which proposes that indigenous populations that had no previous contact with certain pathogens, upon first exposure would be highly susceptible to disease due to lack of preestablished immunity, is unsubstantiated by scientific evidence (21, 22). Beyond genetics, other factors exert a significant effect over the health of indigenous population in Brazil, such as economy, geographical location, contact with non-indigenous people, sanitation and access to basic health assistance (4, 23). Comparisons among Indigenous populations from different Brazilian regions showed that Indigenous people from remote areas, such as the Amazon, were affected by the COVID-19 pandemic to a greater extent than populations located in the more urbanized northeast region (16). Several factors must be considered when interpreting and comparing seroconversion rates in any vaccinated human population, including the number of vaccine doses administered, the time interval between vaccination and sample collection, the mean age of the study population, and prior exposure to the virus. If considering only Brazil, the genetic variability among indigenous and non-indigenous populations would be considerable, meaning our findings do not necessarily reflect the seroconversion rates expected for any other population, even if immunization conditions were exactly matched. Regarding our study population, the Tupiniquim population maintains contact with non-indigenous communities, in contrast with the reclusive Guarani-Mbyá, which may have contributed to greater hospitalization rates observed in Tupiniquim individuals.

Up to June 2021, vaccination coverage was 74.0% among total indigenous populations in Brazil and 94% in Minas Gerais and ES states (8). After the beginning of the vaccination in the present study, from 386 individuals, 379 was vaccinated (98.2%). Therefore, vaccination coverage in our studied populations was higher than the Brazilian average and the SIHDs (Special Indigenous Health Districts) encompassing Minas Gerais and ES states.

We conclude that most vaccinated individuals seroconverted against SARS-CoV-2 antigens, indicating that Coronavac may be as protective in individuals from Tupiniquim and Guarani-Mbyá groups as observed in the general Brazilian population. COVID-19 severity was minimal regardless of incomplete vaccine coverage, suggesting that vaccination may not be the only factor protecting individuals from severe COVID-19. Tuberculosis is highly prevalent in the population studied and was not associated with increased seroconversion to SARS-CoV-2.

## Data availability statement

The original contributions presented in the study are included in the article/**Supplementary Material**. Further inquiries can be directed to the corresponding authors.

## Ethics statement

The study followed the ethical protocols of the responsible committee on human experimentation and was approved by the ethics committees from the National Research Ethics Committee (CONEP, acronym in Portuguese, no 4599) and the Federal University of Espírito Santo. The participants provided their written informed consent to participate in this study.

## Author contributions

AN: Data curation, Formal analysis, Investigation, Methodology, Validation, Writing – original draft. RL: Data curation, Formal analysis, Methodology, Writing – review & editing. JM: Funding acquisition, Resources, Supervision, Writing – review & editing. AP: Investigation, Writing – review & editing. RM: Conceptualization, Investigation, Supervision, Writing – review & editing. TH: Conceptualization, Funding acquisition, Investigation, Project administration, Resources, Supervision, Writing – review & editing.
