# Supplementary material for: The seroconversion history to SARS-CoV-2 in Indigenous people from Brazil – the interplay between exposure, vaccination, and tuberculosis
Source: Front Immunol. 2024 Jul 16;15:1359066. doi: 10.3389/fimmu.2024.1359066 (PMC11286483; doi:10.3389/fimmu.2024.1359066)
Supplement: Supplementary file 1 [file DataSheet_1.pdf]

# *Supplementary Material*

## 1 Supplementary Figures

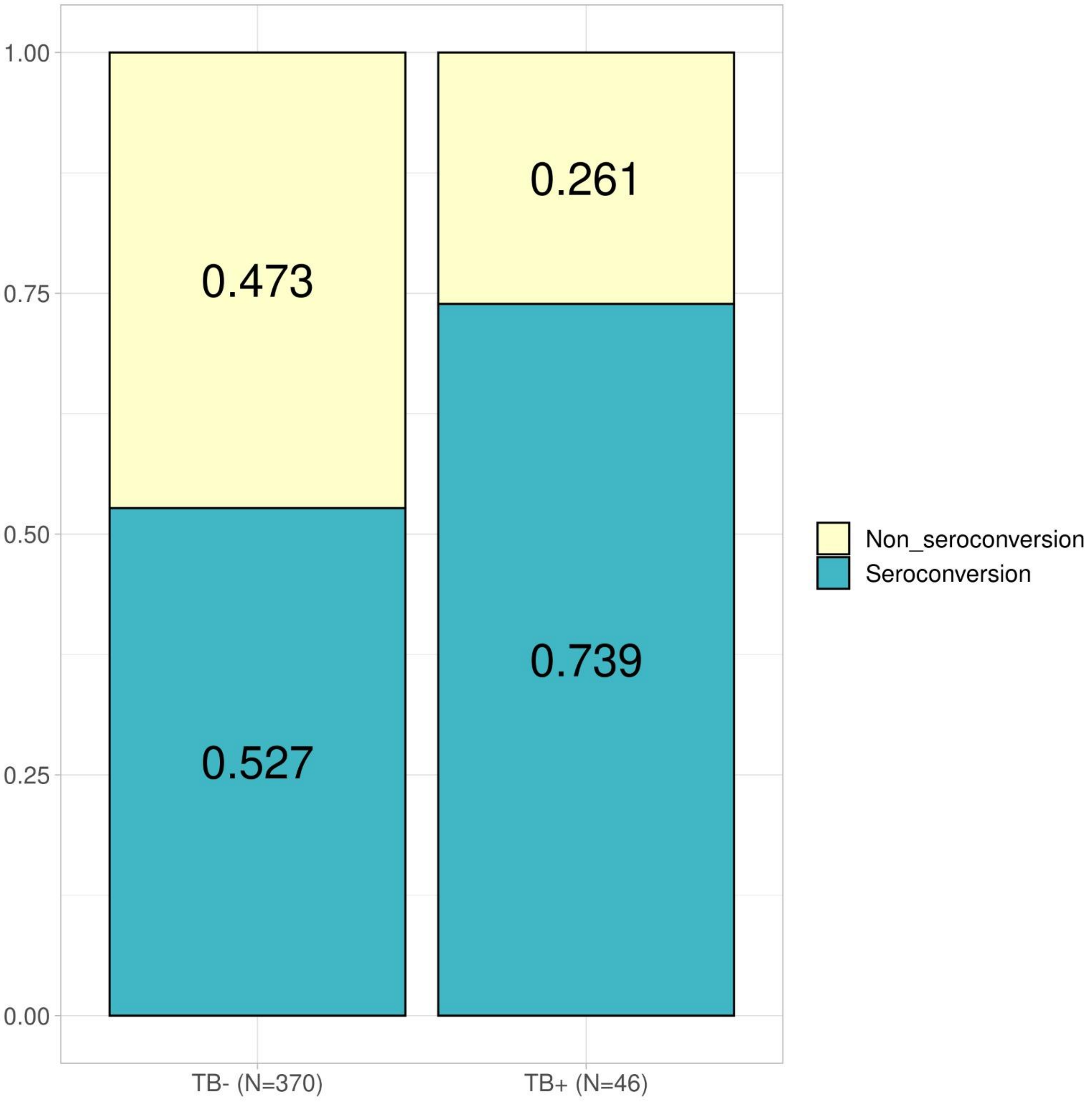
1.00
0.75
0.50
0.25
0.00
0.473
0.527
0.261
0.739
TB- (N=370)
TB+ (N=46)
Non_seroconversion
Seroconversion

**Supplementary Figure 1.** Relationship between results for tuberculosis and seroconversion to SARS-CoV-2. Bar charts showing the proportions of seroconversion to SARS-CoV-2 (represented in blue) and non seroconversion (represented in light pink) among individuals with tuberculosis (on the right) and among individuals without tuberculosis (on the left). The percentages of seroconversion and non seroconversion were calculated in relation to the total number of TB+ (n=46) for the chart on the right and in relation to the total number of TB- (n=370) for the chart on the left.

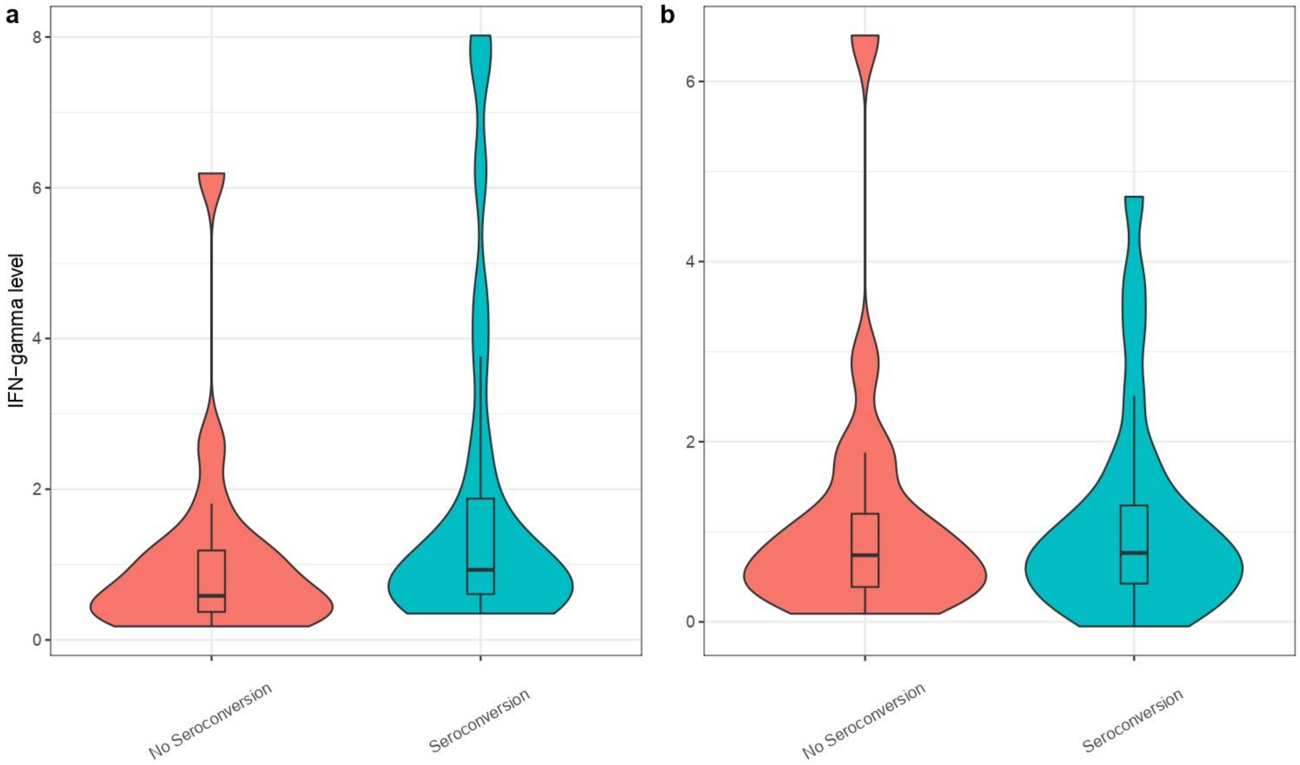


**Supplementary Figure 2.** Differences in the content of IFN-γ (IU/mL) produced in response to antigens ESAT-6 and CFP-10 simulating mycobacterial proteins in people who seroconverted to SARS-CoV-2 and in non-seroconverting people. (A) Quantification obtained from the TB1 tube (CD4+). (B) Quantification obtained from the TB2 tube (CD8+). Individuals with values for TB1 and

TB2 higher than 10 IU/mL were excluded from the analysis because these values are outside the linear range of the assay.

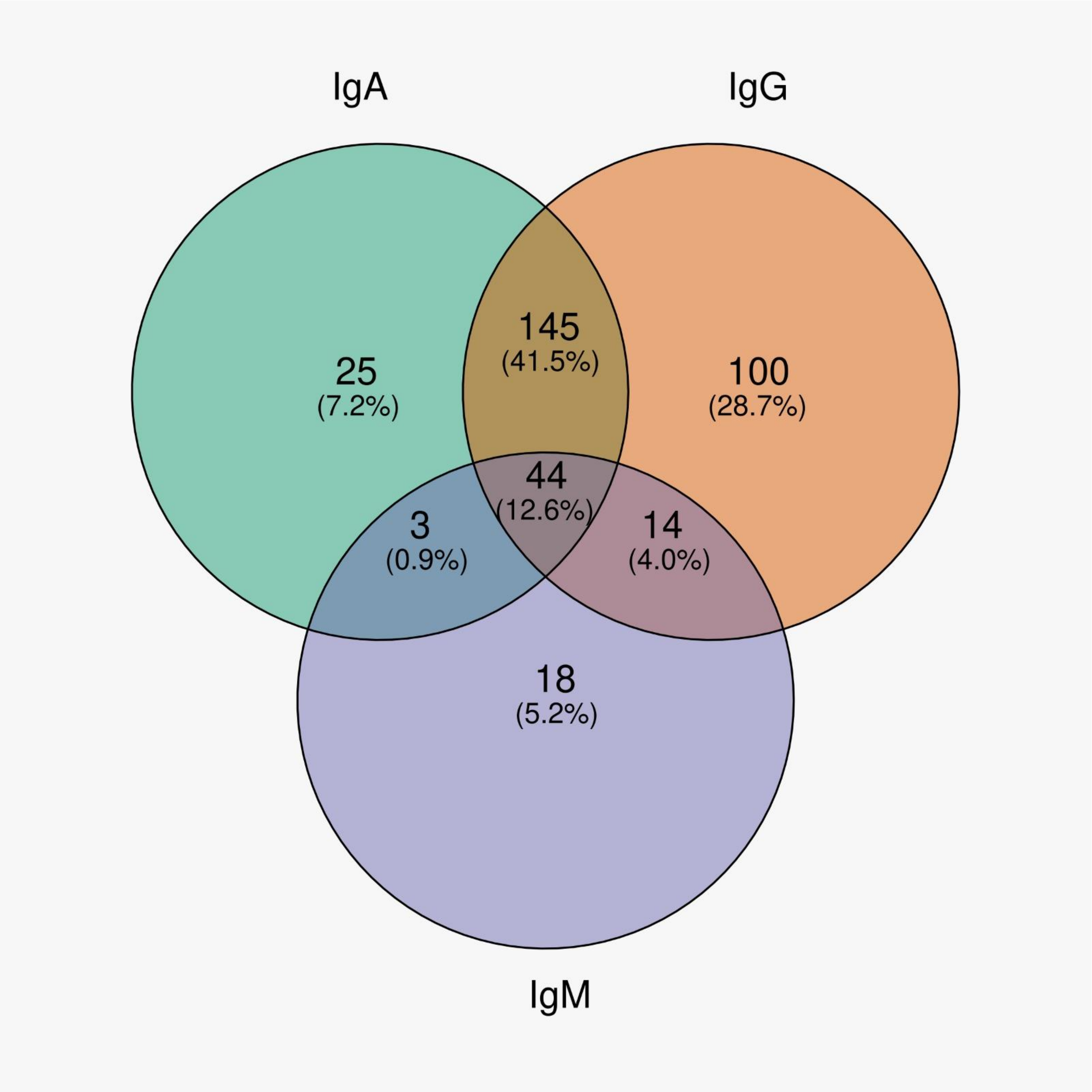


**Supplementary Figure 3.** Venn diagram representing the raw values and the related percentage of people seroconverting to only IgA (green), IgG (salmon) or IgM (purple), or in combination of two or more antibodies. The percentages were calculated in relation to the total seroconversion (n=349).
